# Demographic, clinical, and paraclinical features of patients operated with the diagnosis of acute descending necrotizing mediastinitis: a retrospective study in Southern Iran

**DOI:** 10.1186/s13019-023-02416-w

**Published:** 2023-12-08

**Authors:** Keivan Ranjbar, Reza Shahriarirad, Kamyar Ebrahimi, Armin Amirian, Mohamadreza Karoobi, Parviz Mardani, Amirhossein Erfani, Mohammad Javad Fallahi, Farzaneh Ketabchi, Bizhan Ziaian

**Affiliations:** 1 Thoracic and Vascular Surgery Research Center, Shiraz University of Medical Science, Shiraz, Iran; 2 Student Research Committee, Shiraz University of Medical Sciences, Shiraz, Iran; 3 Department of Surgery, Shiraz University of Medical Sciences, Shiraz, Iran; 4 Department of Physiology, School of Medicine, Shiraz University of Medical Sciences, Shiraz, Iran; 5 Department of Internal Medicine, Shiraz University of Medical Sciences, Shiraz, Iran

**Keywords:** Mediastinitis, Retrospective studies, Reoperation, Sternotomy, Thoracotomy

## Abstract

**Introduction:**

Descending necrotizing mediastinitis (DNM) is a type of acute mediastinitis that is rarely reported but is regarded as a fatal disease despite improvements in technological methods and antibiotic therapies. We aimed to determine the demographic, clinical, and paraclinical features of patients diagnosed with acute DNM.

**Methods:**

In this retrospective study, patients’ hospital records with a diagnosis of DNM admitted to the Namazi hospital in southern Iran during 18 years (2002–2019) were reviewed. Demographic and clinical features were recorded and subsequently analyzed via SPSS 22.

**Results:**

Out of 67 mediastinitis patients, 25 (37.3%) were diagnosed as DNM with an average age of 37.2 ± 16.7 years, and 68% were male. Regarding etiology, 52.0% were due to neck infection. Based on the technique of surgery, 52% of the patients underwent the combined method, which was mostly among type I and IIA DNM, while thoracotomy was mostly performed on type IIB DNM (P = 0.08). Based on the incision, type IIA and IIB had the highest frequency of thoracotomy and cervicothoracic incisions (P = 0.02 and 0.002). Puss discharge was significantly lower in type I DNM (P = 0.01). Based on the presenting symptoms of our patients, the majority (72.0%) had a chief complaint of neck pain, followed by chills and fever (48%). There were no reports of mortality during our short-term follow-up.

**Conclusion:**

We report one of the largest retrospective studies of DNM patients in our referral center, with a high prevalence of the disease among younger populations, especially under 40 years. The method of treatment should be chosen based on the extent of infection and can be limited to neck exploration in upper mediastinal infections, though thoracic or combined approach in more broad infections.

## Introduction

Descending necrotizing mediastinitis (DNM) is a rare but fatal type of acute mediastinitis resulting from the downward extension of infection via the fascial plane and causing necrosis [1]. The precise incidence and prevalence of total mediastinitis cases, including DNM and fibrosing mediastinitis, remain undocumented. However, postoperative mediastinitis is relatively rare, with a frequency ranging from 0.3 to 5% and an average occurrence of 1–2% in most medical facilities [2]. Most patients may have a history of an upper respiratory tract infection, recent dental infection. Although the most common symptoms are dysphagia, neck pain or edema, sore throat and pleuritic or retrosternal chest pain, some patients present with chills, fever, odynophagia, cough, and respiratory distress. Pathogens responsible for DNM are usually aerobic polymicrobial, mainly Gram positives and mixed anaerobic bacteria [3].

The key components of treating DNM include prompt diagnostic imaging, airway management, administration of intravenous antibiotics, and surgical drainage. When it comes to diagnosing mediastinitis, a chest radiograph may provide some assistance if there are indications of mediastinal widening or pneumomediastinum. However, it may not always accurately convey the extent of the disease. On the other hand, computed tomography and magnetic resonance imaging are more effective diagnostic tools for evaluating mediastinitis [4]. Multidisciplinary management is essential for treating DNM, and surgical debridement is a crucial aspect [5]. However, the extent of surgical debridement, whether only transcervical or routine transthoracic thoracotomy, has been a matter of controversy. Surgical and broad-spectrum antibiotic therapies are considered the main treatment of the disease as many studies showed lower mortality rates in early combined and aggressive use of antibiotics and surgeries [1, 6, 7]. While there is limited evidence of successful medication therapy for DNM, many current treatment strategies involve the use of immunosuppressants, corticosteroids, or antifungals due to the limited treatment options available for these patients [8]. Patients with symptoms may benefit from surgical interventions, such as stents or bypasses of the affected structure, to alleviate compression, and these options should be considered [9, 10].

The clinical presentation of DNM is usually mild and lacks specificity, which makes it challenging to diagnose early and often leads to a delayed diagnosis and eventually possible death. As the infection rapidly spreads, delayed diagnosis and treatment may result in a high mortality rate. The mortality rate for DNM used to be as high as 60–70%, but with active surgical management, it has significantly decreased to 30–40% [11]. Nonetheless, a study conducted by Sarna et al. in 2012 discovered that the mortality rate for DNM patients with septic shock remained exceedingly high at 64% [11]. A positive prognosis is typically associated with prompt recognition, early diagnosis, and timely treatment.

DNM is still regarded as a fatal disease despite improvements in technological methods and antibiotic therapies. This retrospective study aimed to determine the demographic, clinical, and paraclinical features of patients diagnosed with acute DNM at Namazi hospital in Southern Iran from 2002 to 2019.

## Method

### Study design and data collection

In this retrospective study, patients’ records that were operated on due to mediastinitis in Namazi hospital in southern Iran during an 18-year period (2002–2019) were evaluated. Data collection was performed retrospectively by referring to the Namazi hospital archive using International Classification of Diseases version 10 (ICD-10) codes to classify cases of mediastinitis. Specifically, we used the code J98.5, which is used to classify diseases of the respiratory system, including inflammation of the mediastinum [12]. To ensure a representative sample of patients with mediastinitis, we used a census sampling method to identify all patients who were operated on due to mediastinitis during the study period. Among those, patients who fulfilled the criteria for DNM were included in our study. The exclusion criteria for this study were patients with mediastinitis originating from an esophageal perforation or any primary infections of the mediastinum. Information related to DNM includes demographics (age, sex), clinical features (such as symptoms and etiology), sources of infection (mandibular, periodontal, necrotizing thyroiditis), extension and location of the infection, type of operation and incision, patient’s outcome (reoperation and mortality), complications, and imaging results were extracted and registered on prepared forms. The infection extension into the mediastinum was assessed via Computed tomography (CT) scans and classified based on Endo’s criteria [13]. Surgery was performed via neck exploration or thoracotomy, or a combination of both. Neck exploration was performed either unilateral or bilateral, through an oblique incision. Lateral or posterolateral thoracotomy was performed in a unilateral or bilateral manner based on the case and complication [14]. Patients were also visited in the clinic during their follow-ups, and any cases of reoperation and mortality were documented.

### Diagnostic and classification of DNM

Estrera et al. in 1983 defined the diagnostic criteria of DNM, which include: clinical manifestations of severe cervical infection, demonstration of characteristic roentgenographic features of mediastinitis, documentation of necrotizing mediastinal infection at operation or postmortem examination, or both, and establishment of a relationship between oropharyngeal infection and development of the necrotizing mediastinal process [15].

In our study, we used Endo’s et al. and also Sergio et al. criteria for the classification of mediastinitis [13, 16]. Type I (focal mediastinitis): Infection found in the superior mediastinal space, on top of bifurcation of the trachea); Type II (diffuse mediastinitis): type II mediastinitis was in turn subdivided into two subtypes: subtype IIA (infection still located in the anterior inferior mediastinal space) and subtype IIB (infection process has reached inferior posterior mediastinum).

### Data analysis

Statistical Package for the Social Sciences (SPSS) software version 22 (IBM Corporation, Armonk, NY) was used to analyze the data. Data were first evaluated for normality by the Kolmogorov-Smirnov normality test. After confirmation of the parameters’ normal distribution, the relationship between DNM and related independent factors was evaluated using either independent sample students’ t-test and analysis of variance (ANOVA) test for continuous, or chi-square (X^2^) or Fisher’s exact test for categorical variables. Descriptive statistics were reported as frequency, percentage (%), mean data distribution, and standard deviation (SD). A P-value of less than 0.05 was considered statistically significant.

## Results

During the period of our study (2002–2019), a total of 67 cases of mediastinitis were operated, of which 23 (34.3%) had an esophageal rupture. Based on the mentioned diagnosis criteria for DNM, 25 (37.3%) patients among a total of 67 mediastinitis patients were diagnosed as acute DNM. The age of the participants ranged from 15 to 77 with an average of 37.2 ± 16.7 years, while 24% were among the age group of 19 to 25 years old. Also, the majority of our cases (n = 17; 68%) were male. A summary of the patients in our study is provided in Table 1.

**Table 1:** Features of descending necrotizing mediastinitis patients

Case	Age/Sex	Surgical procedure	Drainage	Chest tube	Clinical features	Etiology	DNM Type*	Other Features
1	47/M	Neck Exploration	Yes	No	Neck pain	Mandibular Abscess	IIA	Anterior mediastinum abscess especially on the left side (CT)
2	15/F	Neck Exploration	No	No		Neck Infections	I	
3	25/F	Neck Exploration	Yes	No	Neck pain, Chest pain, Fever, Tachycardia, Tachypnea	Periodontal	I	Neck abscess extending to retrosternal space above the level of carina (CT)
4	30/M	Combined	Yes	Yes	Neck pain, Fever, Dysphagia, Tachycardia, Tachypnea	Necrotizing Thyroiditis	IIB	Necrotizing of the parathyroid gland
5	35/M	Combined	Yes	Yes	Neck pain, Dysphagia, Fever, Dyspnea	Neck Infections	IIB	Significant necrosis, developed post operative pleural effusion and sepsis
6	34/M	Neck Exploration	No	No	Neck pain, Chest pain	Neck Infections	I	Ludwig abscess
7	55/F	Combined	No	Yes	Neck pain, Fever, Dyspnea, Decreased LOC	Mandibular Abscess + Periodontal	IIA	Opaque area in the left anterior-inferior mediastinum (CT)
8	17/F	Combined	Yes	Yes	Neck pain, Fever, Dysphagia	Neck Infections	IIA	Developed empyema + Two purulent collection in left thoracic cavity in periaortic and lower pleural cavity
9	23/M	Combined	No	Yes	Chest pain, Fever, Dyspnea, Tachycardia, Tachypnea	Neck Infections	IIA	
10	45/M	Combined	No	Yes	Chest pain, Fever, Dyspnea, Dysphagia	Neck Infections	IIB	Multiloculated right and left side pleural effusion (CT) + Mediastinal widening (CXR)
11	25/M	Neck Exploration	Yes	No		Neck Infections	I	
12	45/M	Combined	No	Yes	Neck pain, Fever	Mandibular Abscess + Periodontal	I	
13	40/M	Combined	No	No	Fever, Dyspnea	Mandibular Abscess + Periodontal	IIA	
14	37/F	Thoracotomy	No	Yes	Neck pain	Neck Infections	I	Bilateral loculated empyema (CT)
15	17/M	Combined	No	Yes	Neck pain, Fever, Tachycardia, Tachypnea	Periodontal	IIB	
16	23/M	Neck Exploration	No	No	Neck pain, Dysphagia	Neck Infections	IIC	
17	53/F	Neck Exploration	No	No	Neck pain, Chest pain	Periodontal	IIC	
18	28/M	Combined	No	Yes	Neck pain	Neck Infections	IIA	
19	41/M	Combined	Yes	Yes	Neck pain, Chest pain, Dyspnea	Neck Infections	IIA	
20	56/M	Combined	No	Yes	Dysphagia	Neck Infections	IIA	
21	22/M	Combined	No	Yes	Fever, Decreased LOC	Periodontal	I	
22	69/F	Neck Exploration	Yes	No	Neck pain, Fever, Tachycardia	Periodontal	IIA	Multiple neck abscesses with involvement of upper anterior mediastinum (CT)
23	19/M	Neck Exploration	Yes	No	Neck pain	Periodontal	I	
24	51/F	Combined	No	Yes	Neck pain	Neck Infections	I	
25	77/M	Combined	No	Yes	Neck pain, Chest pain	Neck Infections	IIC	Neck abscess extending to retrosternal space above the level of carina (CT); Ludwig angina

CT: Computed tomography; CXR: Chest X-ray; DNM: Descending necrotizing mediastinitis; F: Female; LOC: level of conciseness; M: Male

* Type based on Sergio classification

There has been an increase in the number of cases throughout the years, from an annual rate of one case per year from 2002 to 2007, and also from 2008 to 2013, and finally a rate of 2.17 cases per year from 2014 to 2019.

Among the 25 patients, two underwent reoperation due to DNM complications (Case 5 and 8). One of our cases (Case 5) initially presented with type I DNM and was operated via neck exploration, in which during operation inflammation of neck and mediastinum and abscess formation in mediastinum (about 30 cc pus) was observed. Mediastinotomy and drainage of the mediastinal abscess, along with 100 cc turbid fluid in right chest tube and 300 cc turbid fluid in left chest tube was performed. The wound was left open along with proper dressing; however, the patient developed pleural effusion and sepsis, so bilateral thoracotomy and drainage of mediastinum was performed, which revealed necrotizing mediastinitis especially in left side and about 100 cc pus in posterior mediastinum in paraesophageal plane.

Among the performed operations and based on the Sergio classification, 9 (36.0%) were type I, 9 (36.0%) were type IIA, 4 (16.0%) were type IIB, and 3 (12.0%) were type IIC DNM. There was no significant association between the type of DNM and etiology (P = 0.85), age (P = 0.20). Table 2 demonstrated the demographical and clinical features of operations for DNM in our study.

**Table 2:** Demographical and clinical features of operations due to acute descending necrotizing mediastinitis from 2002 till 2019 (N = 28)

Variable		Total (%); N = 25	Type				P-value
			**I**; *n = 9*	**IIA**; *n = 9*	**IIB**; *n = 4*	**IIC**; n = 3	
Year; n (%)	2002–2007	6 (24.0)	2 (33.3)	2 (33/3)	2 (33.3)	0 (0)	0.48
	2008–2013	6 (24.0)	2 (33.3)	4 (66.7)	0 (0)	0 (0)	
	2014–2019	13 (52.0)	5 (38.5)	3 (23.1)	2 (15.4)	3 (23.1)	
Gender; n (%)	Male	17 (68.0)	5 (29.4)	6 (35.3)	4 (23.5)	2 (11.8)	0.54
	Female	8 (32.0)	4 (50.0)	3 (37.5)	0 (0)	1 (12.5)	
Age (years); mean ± SD		37.2 ± 16.7	30.3 ± 12.2	41.8 ± 17.0	31.8 ± 11.6	51.0 ± 27.1	0.20
Age Group (years); n (%)	≤ 18	3 (12.0)	1 (33.3)	1 (33.3)	1 (33.3)	0 (0)	0.69
	19–25	6 (24.0)	4 (66.7)	1 (16.7)	0 (0)	1 (16.7)	
	26–35	4 (16.0)	1 (25.0)	1 (25.0)	2 (50.0)	0 (0)	
	36–45	5 (20.0)	2 (40.0)	2 (40.0)	1 (20.0)	0 (0)	
	46–55	4 (16.0)	1 (25.0)	2 (50.0)	0 (0)	1 (25.0)	
	> 55	3 (12.0)	0 (0)	2 (66.7)	0 (0)	1 (33.3)	
Necrotic Tissue; n (%)		7 (28.0)	2 (28.6)	3 (42.9)	2 (28.6)	0 (0)	0.70
Etiology; n (%)	Neck infection	13 (52.0)	4 (30.8)	5 (38.5)	2 (15.4)	2 (15.4)	1.00
	Periodontal	10 (40.0)	4 (40.0)	4 (40.0)	1 (10.0)	1 (10.0)	1.00
	Mandibular Abscess	5 (20.0)	2 (40.0)	3 (60.0)	0 (0)	0 (0)	0.68
Surgical Procedure; n (%)	Neck Exploration	9 (36.0)	5 (55.6)	2 (22.2)	0 (0)	2 (22.2)	**0.08**
	Thoracotomy	3 (12.0)	1 (33.3)	0 (0)	2 (66.7)	0 (0)	
	Combined	13 (52.0)	3 (23.1)	7 (53.8)	2 (15.4)	1 (7.7)	
Type of Operation; n (%)	Neck Exploration	20 (80.0)	8 (40.0)	7 (35.0)	2 (10.0)	3 (15.0)	0.37
	Thoracotomy	16 (64.0)	4 (25.0)	7 (43.8)	4 (25.0)	1 (6.3)	0.16
	Chest tube insertion	15 (60.0)	4 (26.7)	6 (40.0)	4 (26.7)	1 (6.7)	0.22
	Drain Insertion	9 (36.0)	3 (33.3)	4 (44.4)	2 (22.2)	0 (0)	0.69
	Mediastinum Exploration	8 (32.0)	3 (37.5)	3 (37.5)	2 (25.0)	0 (0)	0.72
	Neck Vessel Exploration	4 (16.0)	2 (50.0)	0 (0)	0 (0)	2 (50.0)	**0.04**
	Pneumolysis	2 (8.0)	0 (0)	0 (0)	2 (50.0)	0 (0)	**0.03**
	Sternotomy	2 (8.0)	1 (50.0)	1 (50.0)	0 (0)	0 (0)	1.00
	Decortication	2 (8.0)	0 (0)	0 (0)	2 (100)	0 (0)	**0.03**
	Pleurotomy	1 (4.0)	0 (0)	0 (0)	1 (100)	0 (0)	0.28
Symptom; n (%)	Neck pain	18 (72.0)	6 (33.3)	6 (33.3)	3 (16.7)	3 (16.7)	0.85
	Chills/Fever	12 (48.0)	3 (25.0)	5 (41.7)	4 (33.3)	0 (0)	**0.04**
	Chest pain	7 (28.0)	2 (28.6)	2 (28.6)	1 (14.3)	2 (28.6)	0.55
	Dysphagia	6 (24.0)	0 (0)	2 (33.3)	3 (50.0)	1 (16.7)	**0.03**
	Dyspnea	6 (24.0)	0 (0)	4 (66.7)	2 (33.3)	0 (0)	0.05
	Tachycardia	5 (20.0)	1 (20.0)	2 (40.0)	2 (40.0)	0 (0)	0.37
	Tachypnea	4 (16.0)	1 (25.0)	1 (25.0)	2 (50.0)	0 (0)	0.34
	Decreases level of conciseness	2 (8.0)	1 (50.0)	1 (50.0)	0 (0)	0 (0)	1.00
	Cough	2 (8.0)	1 (50)	1 (50)	0 (0)	0 (0)	1.00
Puss discharge; n (%)		13 (52.0)	1 (7.7)	6 (46.2)	3 (23.1)	3 (23.1)	**0.01**
Incision; n (%)	Cervical	20 (80.0)	7 (35.0)	8 (40.0)	2 (10.0)	3 (15.0)	0.37
	Thoracotomy	14 (56.0)	2 (14.3)	7 (50.0)	4 (28.6)	1 (7.1)	**0.02**
	Cervicothoracic	13 (52.0)	1 (7.7)	7 (53.8)	4 (30.8)	1 (7.7)	**0.002**
	Superficial	1 (3.6)	0 (0)	1 (100)	0 (0)	0 (0)	1.00
Incision Position; n (%)	Classic cervical	20 (80.0)	7 (35.0)	8 (40.0)	2 (10.0)	3 (15.0)	0.37
	Right posterolateral thoracotomy	5 (20)	0 (0)	2 (40.0)	2 (40.0)	1 (20.0)	0.13
	Left posterolateral Thoracotomy	1 (100)	0 (0)	0 (0)	1 (100)	0 (0)	0.28
	Right anterolateral thoracotomy	5 (20)	1 (20.0)	2 (40.0)	2 (40.0)	0 (0)	0.37
	Left anterolateral thoracotomy	3 (12.0)	1 (33.3)	1 (33.3)	1 (33.3)	0 (0)	1.00
	Oblique anterior and parallel to sternocleidomastoid muscle	14 (56.0)	4 (28.6)	6 (42.9)	1 (7.1)	3 (21.4)	0.25

Based on the presenting symptoms of our patients, the majority (72.0%) had a chief complaint of neck pain, followed by chills and fever (48%), chest pain (28%), and dysphagia (24%). Dysphagia was significantly lower among type I patients (P = 0.03). There were no reports of mortality during our short-term follow-up.

Among the etiologies of DNM, the majority were due to neck infection (52.0%), followed by periodontal infection (40.0%) and mandibular abscess (20.0%). Three cases (cases 7,12, and 13) had both mandibular abscess and periodontal infection. There was no statistical difference among the etiological groups in terms of age (P = 0.58), or gender (P = 0.61).

Based on the technique of surgery, 52% of the patients underwent combined method, which was mostly among type I and IIA DNM, while thoracotomy was mostly performed on type IIB DNM (P = 0.08). Based on the incision, type IIA and IIB had the highest frequency of thoracotomy and cervicothoracic incisions (P = 0.02 and 0.002). Puss discharge was significantly lower in type I DNM (P = 0.01). There was no significant association between the method of surgery and the patients age (P = 73), gender (P = 0.71), patients’ symptoms and etiologies.

## Discussion

We report results of a retrospective study in a referral center during an 18-year period, in which a total of 25 patients with DNM were operated. Our study is among the largest reports of DNM cases among available literature. The age of the participants ranged from 15 to 77 with an average of 37.2 ± 16.7 years, while the majority (24%) were among the age group of 19 to 25 years old. Our data were contrary to previous reports, which demonstrated that patients in their 60s were the age group with the highest number of DNM patients (28%), while 29.8% of the patients were in their 40 and 50 s, and 5.8% were under the age of 40. [16–20] Congedo et al. also reported that the median age of DNM patients was 43 among males and 63 among females [21]. However, all reports relatively demonstrate a male dominance in this entity [22]. Nevertheless, due to the relative rarity of this entity, data in this regard are scattered, making the diagnosis and management of the disease a challenge for physicians and surgeons worldwide.

Based on the presenting symptoms of our patients, the majority (71.4%) had a chief complaint of neck pain, followed by chills and fever (50%), dysphagia (28.6%), and chest pain (25%). Also, all cases of dysphagia were among neck infection patients. Similar to our study, all participants in a study by Wei et al. [23] had cervical discomfort. Some had soft tissue edema and inflammation in the lower cervical and upper thoracic regions. In their study, fever, increasing chest discomfort, jugular vein distension, and shortness of breath were common symptoms in DNM patients. [23] DNM, however, is difficult to distinguish from deep neck infection based only on clinical manifestations, which may explain the gap between diagnosis and treatment. DNM following deep neck infection is usually caused by the spreading of cellulitis or abscesses in the fascial planes and potential spaces of the neck to the chest [20]. In our study, the most frequent etiology was neck infection (57%), followed by periodontal infection (32.1%) and mandibular abscess (14.3%). We observed no significant association between the etiologies and the type of DNM. A study by Ho et al. in Taiwan reported that 71.6% of patients with deep neck infection were type I DNM, while in our study only 30.8% of type I DNM were due to neck infection [22, 24]. In Western countries, acute mediastinitis caused by primary oropharyngeal or odontogenic infection is now uncommon. At the same time, this disease is more common in developing countries due to poor economic conditions and a lack of medical resources for dental and oropharyngeal disease prevention and treatment. According to a report from Taiwan, this lethal complication will develop in about 2.6% of patients who have a deep neck infection. [25, 26] In an 18-year period, our institution treated 25 patients, indicating that the occurrence is not uncommon. However, the increase in the number of cases throughout the years might be due to the improvement of detection methods and prompt diagnosis and management. Nevertheless, DNM is among diseases with high morbidity and mortality rates. [27]

Efforts to reduce the mortality rate associated with DNM have only been moderately successful over the last 50 years. Because DNM frequently has a fulminant course, the high fatality rate is due to a delay in diagnosis [14, 28–38]. Pearse [39] reported that 49% of patients with DNM died during treatment in the first modern series published in 1938. In our study, we observed no cases of mortality, which could be due to the fact that our province has multiple referral centers with excellent medical services which could aid in early identification. However, despite the subsequent introduction of intravenous antibiotics, vast improvements in anesthesia and critical care, and the development of CT imaging, the death rate for patients with DNM reported in the literature over the last three decades has remained high [15, 28, 38].

Prior to the 21st century, the main methods for surgery were the trans-thoracic (37%) or transcervical (54%), while the combined method was only performed in 2% of the cases. These techniques were accompanied by high mortality (32%), especially in cases with progressed disease; however, a decrease in mortality rate to 18% was achieved through early combined thoracic-cervical drainage [1, 40–42]. Also, some authors preferred sternotomy or clamshell incision based on the good access to both thoracic cavities and the anterior mediastinum [17, 43, 44]. This technique was only used in two of our patients in our study. Even when done in single-lung ventilation, this method seems less suitable for draining the infero-posterior mediastinum collections, aside from the potential for osteomyelitis and sternal dehiscence [21].

The stage and degree of DNM, as well as the clinical status of the patients, should be carefully considered when operational approaches are chosen. Based on a systematic review of 26 reports of DNM worldwide, among 480 cases, 189 (39.4%) were type I while 249 (51.9%) were type II [1]. These results are in line with our study, in which 36% were type I while 64% were type II DNM. Neck exploration with transcervical mediastinal drainage may be sufficient when type I DNM is present. [37] Congeto et al. stated that in managing patients with infection confined in the antero-superior mediastinum region, transcervical mediastinal drainage in sufficient, however, DNM types I and II instead require a thoracic approach, such as Video-Assisted Thoracic surgery or open surgery. They also stated that patients with both anterior and posterior involvement, were treated with bilateral or unilateral thoracic surgery and reported a mortality rate of 27.6%, higher than other groups [21]. Based on our study and Sergio et al. classification, these patients are categorized in the IIB group, which similar to previous studies, underwent either thoracotomy or a combined method of treatment. Based on previous reports, early transcervical mediastinal approach and cervicotomy is the suitable treatment method for limited DNM, especially the upper mediastinum, while thoracotomy was reserved for patients in which the infection extended to below the plane of the tracheal bifurcation [19, 21]. A transcervical drainage may even be sufficient when a thoracic surgeon is not available [41]. The transthoracic method is more invasive compared to the transcervical approach and should be considered in complicated patients with uncontrolled infection [45]. Our study was also in line with previous therapeutic approaches, in which the majority of type I DNM patients underwent neck exploration. Furthermore, De Palma et al. and Congedo et al. advised that in patients with initially limited mediastinitis, a lateral thoracotomy be performed alongside cervicotomy to achieve mediastinal debridement and a toilette of pleural collections [21, 46].

Among the main limitations of our study is the retrospective nature, which included missing data among the patient’s hospital records. Another limitation is the lack of long-term patient follow-up and infecting pathogen evaluation. Further studies are needed to assess the spectrum of clinical presentations and compare the efficiency of various surgical procedures in managing DNM patients. Also, the lack of specific outcome and small number in each classification prevents us from performing statistical analysis and providing risk factors or significant correlations. Therefore, further randomized controlled trials and multicentric studies should be performed to evaluate the most optimum therapeutic approach in managing this rare but lethal entity.

## Conclusion

We report one of the largest case studies regarding DNM patients in our center while also achieving an optimal outcome. Our study demonstrated a high prevalence of the disease among younger populations, especially under 40 years. DNM is an uncommon but serious condition that requires prompt diagnosis and appropriate surgical management. The use of a multi-disciplinary team for standardizing a protocol that offers prompt diagnosis and appropriate surgical management is essential. The method of surgery should be adjusted based on the patient and also the extent of infection, while neck exploration could be considered in type I patients with limited upper infection, while thoracotomy or a combined method is more advised in patients with infection extending to the lower mediastinum. Our study provides valuable information for clinicians and surgeons in the diagnosis and management of this rare but potentially life-threatening condition.

## Data Availability

The datasets generated and/or analyzed during the current study are not publicly available due to patients’ details but are available from the corresponding author on reasonable request.
